# Molecular Mechanism of the Asarum–Angelica Drug Pair in the Treatment of Periodontitis Based on Network Pharmacology and Experimental Verification

**DOI:** 10.3390/ijms242417389

**Published:** 2023-12-12

**Authors:** Qianyang Chen, Yuhan Wang, Chun Shi, Meichen Tong, Haibo Sun, Ming Dong, Shuo Liu, Lina Wang

**Affiliations:** 1 Department of Endodontics and Periodontics, College of Stomatology, Dalian Medical University, 9 West Section, Lvshun South Road, Dalian 116044, China; 2 Academician Laboratory of Immune and Oral Development and Regeneration, Dalian Medical University, Dalian 116044, China

**Keywords:** Asarum–Angelica, drug pair, network pharmacology, docking simulation, periodontitis

## Abstract

(1) To examine the potential mechanism of the Asarum–Angelica drug pair against periodontitis and provide an experimental basis for the treatment of periodontitis with herbal medicine. (2) The core components and core targets of the Asarum–Angelica drug pair in the treatment of periodontitis were detected according to network pharmacology methods. Finally, the effect of the Asarum–Angelica drug pair on osteogenic differentiation was observed in mouse embryonic osteoblast precursor cells. (3) According to the results of network pharmacology, there are 10 potential active ingredients in the Asarum–Angelica drug pair, and 44 potential targets were obtained by mapping the targets with periodontitis treatment. Ten potential active ingredients, such as kaempferol and β-sitosterol, may play a role in treating periodontitis. Cell experiments showed that the Asarum–Angelica drug pair can effectively promote the expression of osteoblast markers alkaline phosphatase (ALP), Runt-related Transcription Factor 2 (RUNX2), and BCL2 mRNA and protein in an inflammatory environment (*p* < 0.05). (4) Network pharmacology effectively analyzed the molecular mechanism of Asarum–Angelica in the treatment of periodontitis, and the Asarum–Angelica drug pair can promote the differentiation of osteoblasts.

## 1. Introduction

Periodontitis is the sixth-most prevalent disease worldwide [1,2]. Studies have shown that periodontal disease is a multifactorial disease dominated by bacteria [3,4], and the protective and destructive mechanism of host immunity is an important link in the progression of periodontal disease [5,6]. However, the pathogenesis of periodontitis is not fully understood and there is currently no effective treatment. Revealing the key factors that induce the immune inflammatory response in periodontitis will provide new evidence for studying the etiology of periodontitis. As a traditional medicine, herbal medicine has good therapeutic effects in many diseases. Herbal medicine also plays a key role in the treatment of COVID-19-related diseases [7,8,9]. Examining the mechanism of Chinese medicine in periodontal disease will provide a new direction for the treatment of periodontitis.

Previous studies have suggested that traditional Chinese medicine has an anti-inflammatory function in periodontitis and simultaneously improves alveolar bone remodeling [10,11,12]. In the clinic, many traditional Chinese medicine formulas for treating periodontitis contain Asarum and Angelica. Asarum comprises approximately 100 plant species, distributed in North America, Europe, and East Asia [13], and is a traditional medicine that has been used as a remedy for toothache in China, Japan, and Korea. Asarum exhibits a broad spectrum of biological effects, such as anti-nociceptive and anti-inflammatory activity [14]. Angelica is both wild and cultivated in China and Korea; it has long been applied in the treatment of inflammation, arthritis, and headaches, and the biological effects of Angelica include neuroprotective, anti-tumor, anti-arthritis, anti-inflammatory, sedative, and immunosuppressive functions [15,16,17]. Based on the biological functions and clinical applications of Asarum and Angelica, Wang et al. [18] employed data mining to investigate the prescription patterns of anti-depressant drugs by traditional Chinese medicine experts and identified the Asa–Ang herb pair as a frequently used core combination in the treatment of paralysis, with further research needed to explain the scientific nature of the combination therapy of the Asarum–Angelica drug pair.

In 2007, Hopkins et al. introduced the concept of “network pharmacology” as a novel approach to drug research and development. This approach involves analyzing the impact of drugs on disease networks and constructing a “drug–target–disease” network. The authors highlighted the significance of this approach in guiding experimental research on the mechanism of action of active ingredients found in traditional Chinese medicine [19,20]. In contrast to conventional pharmacological approaches, network pharmacology incorporates principles from systems biology and multidirectional pharmacology. It combines biological and drug action networks to examine the interplay between drugs and nodes or network modules within the network. This multi-targeting research strategy enables a comprehensive analysis of drug action through a network-based approach. The user’s text does not contain any information to rewrite the approach [21]. Molecular docking technology [22] is the identification of intermolecular interactions and prediction of optimal binding modes of molecules and proteins based on receptors or ligands of known structure, adopting the principle of complementarity, which can provide a theoretical basis for the search and further development of lead compounds [23]. Massive amounts of protein–protein interaction (PPI) data have been mined with the rapid development of high-throughput technologies, providing a data basis for proposing methods for identifying critical proteins in protein interaction networks and computational identification of protein complexes [24].

Based on the Systems pharmacology database and analysis platform of traditional Chinese medicine [25], this study analyzed the possible mechanism of the Asarum–Angelica drug pair in treating periodontitis from the perspective of bioinformatics. In addition, the effect of Asarum–Angelica on the proliferation and differentiation of osteoblasts was validated by cell experiments.

## 2. Results

### 2.1. Active Ingredients and Targets Associated with Periodontitis in the Asarum–Angelica Drug Pair

**Figure 1:**
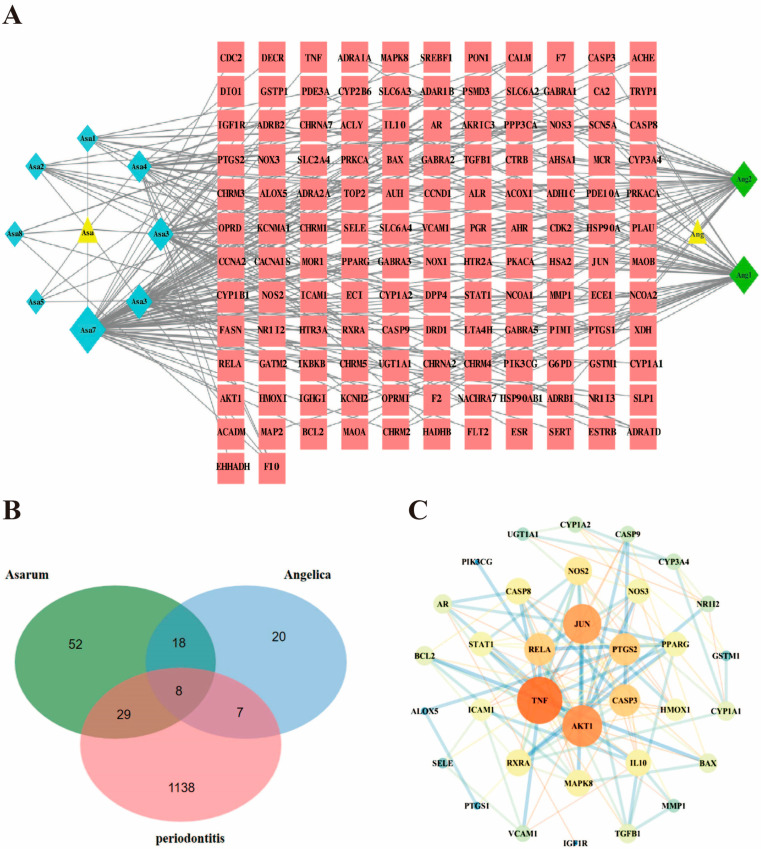
Map of the active ingredients and periodontitis targets of the Asarum–Angelica drug pair. (**A**) Active ingredient target network diagram of the Asarum–Angelica drug pair. Asa represents Asarum, Ang represents Angelica, triangles are drug names, diamonds are drug active ingredients, squares are targets, the area represents the size of the degree value and the corresponding ingredients are specified in Table 1. (**B**) Venn diagram of the Asarum–Angelica drug pair–periodontitis target. (**C**) PPI network diagram of the drug pair–periodontitis target of Asarum–Angelica.

The TCMSP database and literature review initially obtained 327 chemical components of Asarum–Angelica, with 10 active ingredients obtained after screening using the OB and DL parameters, including 8 active ingredients of Asarum and 2 active ingredients of Angelica, including kaempferol, β-sitosterol, and dousterol (Table 1). There were 161 targets for the action of the components of Asarum and 68 targets for the action of the components of Angelica. The target prediction results were combined and duplicate values were removed, and a total of 138 targets were obtained. A total of 146 nodes (containing 134 targets, 10 active ingredients, and 2 drugs) with 239 relationships were obtained using Cytoscape 3.7.1 to map and analyze the relationship network between the active ingredients and their targets of the Asarum–Angelica drug pair, as shown in Figure 1A. A search of the database using the keyword “periodontitis” yielded 1182 periodontitis targets. The screened active ingredient targets of Asarum–Angelica were intersected with the periodontitis targets, and the Wayne diagram was drawn by microbiological letter to obtain 44 common targets of the Asarum–Angelica drug pair components–periodontitis, as shown in Figure 1B. Protein interaction analysis was performed using the STRING 11.0 database on the intersectional targets of the Asarum–Angelica drug pair and periodontitis, and a total of 34 nodes and 124 edges of the intertarget interaction network were obtained by screening. The regulation of periodontitis by the active ingredients of the Asarum–Angelica drug pair may be closely related to the targets of tumor necrotizing factor (TNF), serine/threonine protein kinase (AKT1), jun proto-oncogene, AP-1 transcription factor subunit (JUN), RELA proto-oncogene (RELA), prostaglandin endoperoxide synthase 2 (PTGS2), and Caspase 3 (CASP3). The PPI network diagram is shown in Figure 1C.

### 2.2. Gene Ontology and Kyoto Encyclopedia of Genes and Genomes Pathway Enrichment Analysis

**Figure 2:**
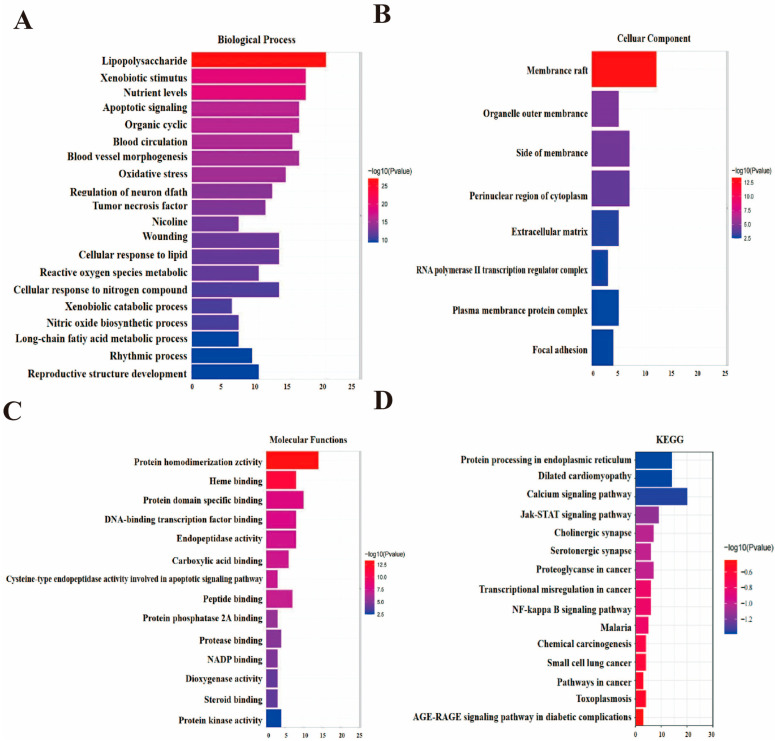
Potential target enrichment analysis of the Asarum–Angelica drug pair on major components. (**A**) GO-BP analysis. (**B**) GO-CC analysis. (**C**) GO-MF analysis. (**D**) KEGG analysis.

Drug–disease intersection targets were imported into the Metascape data platform for signaling pathway analysis of the Asarum–Angelica drug pair regulating periodontitis-related targets. The results suggest that the function of multiple targets in the drug pair is inextricably linked to the development of periodontitis. Biological processes (BP) are mainly regulated in response to LPS, response to xenobiotic stimulus, response to nutrient levels, apoptotic signaling pathway, and response to organic cyclic compounds, as shown in Figure 2A. Cellular components (CC) such as membrane rafts, organelle outer membranes, and extracellular matrix were mainly significantly affected in the Cellular Component (see Figure 2B). The molecular function (MF) is mainly on proton homodimerization activity, heme binding, and DNA-binding transcription factor binding (see Figure 2C). Kyoto Encyclopedia of Genes and Genomes pathway enrichment analysis showed that the signaling pathways involved in the regulation of periodontitis by Asarum–Angelica were mainly the AGE–RAGE signaling pathway, NF-κB signaling pathway, JAK/STAT signaling pathway, small cell lung signaling pathway, etc. (see Figure 2D). The enrichment outcomes of the designated paths are presented in Table 2.

### 2.3. Construction of a Constituent–Periodontitis Target–Pathway Network Diagram for the Asarum–Angelica Drug Pair

**Figure 3:**
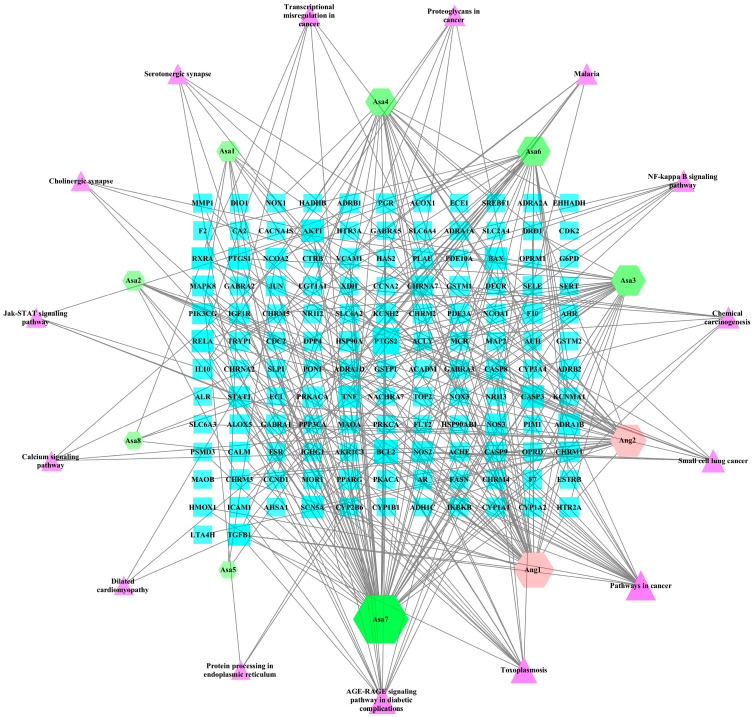
Diagram of the Asarum–Angelica drug pair component–periodontitis target–pathway network. Hexagonal nodes of different colors are active ingredients of different drugs, rectangles are periodontitis targets, and positive triangles are access points. The area and color transparency of the nodes represent their degree; the larger the area and the darker the color, the more important the node is. Asa represents Asarum, Ang represents Angelica.

Using Cytoscape 3.7.1, a network diagram of the constituent–periodontitis target–pathway of the Asarum–Angelica drug pair was constructed, as shown in Figure 3. The degree of kaempferol connectivity (Degree) was 61, the betweenness (Degree) was 0.4137, and the tightness (Closeness) was 0.4788, predicting kaempferol to be the main component of the Asarum–Angelica drug pair in regulating periodontitis, followed by β-sitosterol, dousterol, and cryptophylline. The analysis of the parameters of the drug pair–periodontitis target of Asarum–Angelica is shown in Table 3. The connectivity of PTGS2 in the network was 14, the mediocrity was 0.1672, and the tightness was 0.5015, while the connectivity of BCL2 in the network was 10, the mediocrity was 0.0253, and the tightness was 0.3788. It is predicted that PTGS2 and BCL2 are the most important targets of the Asarum–Angelica drug pair for regulating periodontitis, and AKT1, CASP3, BAX, RELA, etc., are also relatively important targets.

### 2.4. Molecular Docking Verification and Molecular Dynamics Simulation

**Figure 4:**
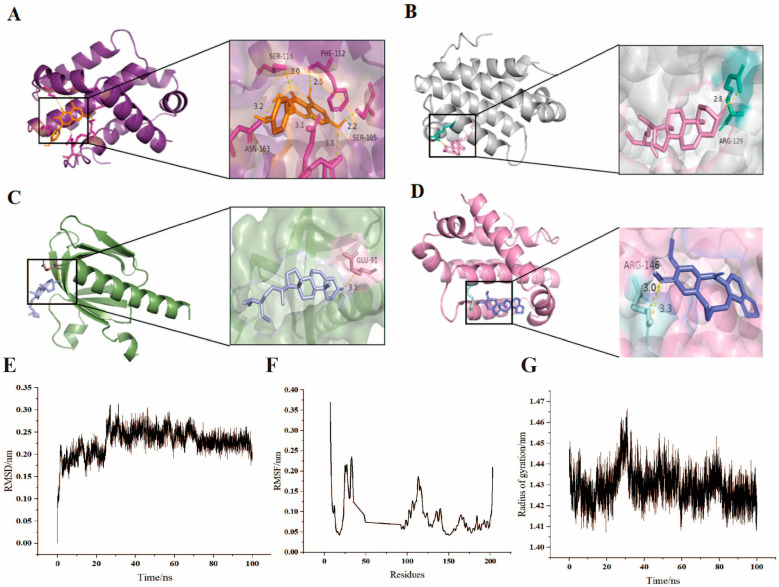
Molecular docking model and molecular dynamics simulation. (**A**) Kaempferol and BCL2. (**B**) Stigmasterol and BCL2. (**C**) β-sitosterol and BCL2. (**D**) Cryptopine and BCL2. (**E**) RMSD line chart. (**F**) RMSF line chart. (**G**) ROG line chart.

BCL2, one of the 13 key targets, was selected for molecular docking with the top four active ingredients in the “Asarum–Angelica Pairs–Active Ingredient Target” network based on Degree analysis using AutoDock Vina software. The processed ligands and receptors were docked using AutoDock Vina software. If the binding energy is <0 kJ∙mol^−1^, then the compound can spontaneously bind to the target protein. The molecular docking results showed that the binding energies of BCL2 to the active ingredients were all less than 0.00 kcal/mol. The molecular docking junctions showed that the binding energy between BCL2 and kaempferol, stigmasterol, β-sitosterol, and cryptopine was −6.7, −8.1, −7.7, and −7.4 kcal/mol, respectively. The molecular docking results of BCL2 with kaempferol, β-sitosterol, dousterol, and cryptopine were visualized and analyzed using PyMOL 2.3 software, and all were found to interact through hydrogen bonds (Figure 4A–D). The most tightly bound combination, BCL2, was selected for molecular dynamics simulations with stigmasterol. RMSD calculates the Root Mean Square Deviation of the backbone and is a valuable parameter for estimating changes or variations in molecular conformation. During the 100 ns of simulation, the RMSD value remained low and smooth (Figure 4E), indicating that the ligand–receptor binding is relatively tight and the complex is stable. Root Mean Square Fluctuation (RMSF) is used to check for regions with high levels of fluctuation. The higher the value, the heavier the interaction of the protein’s residues with small molecules. Based on the observation in Figure 4F, it can be seen that Res25-Res50 and Res107-Res125 have larger RMSF values during the simulation, suggesting that these residues may be critical residues. The radius of gyration (Rg) is the ultimate criterion for deciphering the stable, compact, and folded conformation of a protein. The analysis shows that the radius of gyration of the proteins is smaller than 2 nm during the simulation, indicating that the protein structure is more compact and the complex is more stable (Figure 4G). The results of molecular dynamics simulations suggested that the compounds were tightly bound and the complex was stable, suggesting that modulation of BCL2 may be a potential mechanism of action for the treatment of periodontitis with the Asarum–Angelica drug pair.

### 2.5. The Effect of the Asarum–Angelica Drug Pair on the Proliferation and Differentiation of MC3T3-E1 Cells

**Figure 5:**
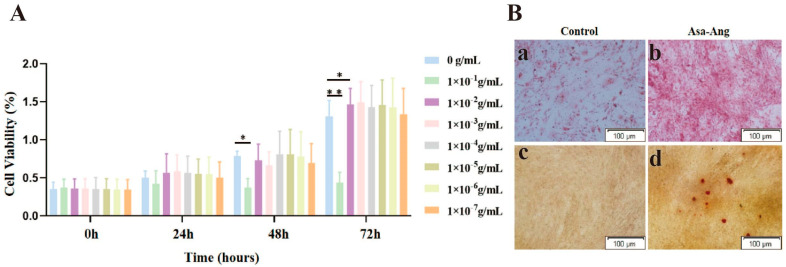
Effect of the Asarum–Angelica drug pair on the proliferation and differentiation of MC3T3-E1 cells. (**A**) CCK-8 staining results (* *p* < 0.05). (**B**) (**a**,**b**): the results of the drug solution acting on MC3T3-E1 cells for 7 days, ALP staining results graph; (**c**,**d**): the results of the drug solution acting on MC3T3-E1 cells for 21 days, alizarin red-staining results graph.

The results of the CCK-8 experiment showed that MC3T3-E1 cells proliferated significantly (*p* < 0.01) at 48 h and 72 h at a drug concentration of 1 × 10^−4^ g/mL compared with the control group, as shown in Figure 5A. The results of ALP staining showed a darker coloration in the intervention group of the Asarum–Angelica drug pair, demonstrating that the Asarum–Angelica drug pair could promote osteogenic differentiation of MC3T3-E1 cells, as shown in Figure 5B(a,b). Consistent with the ALP staining results, the number of calcium nodules was significantly higher in the 21-day alizarin red-stained Asarum–Angelica drug pair group compared to the blank control group, as shown in Figure 5B(c,d).

### 2.6. The Effect of the Asarum–Angelica Drug Pair on Osteogenesis-Related Genes and Protein Expression in MC3T3-E1 Cells

**Figure 6:**
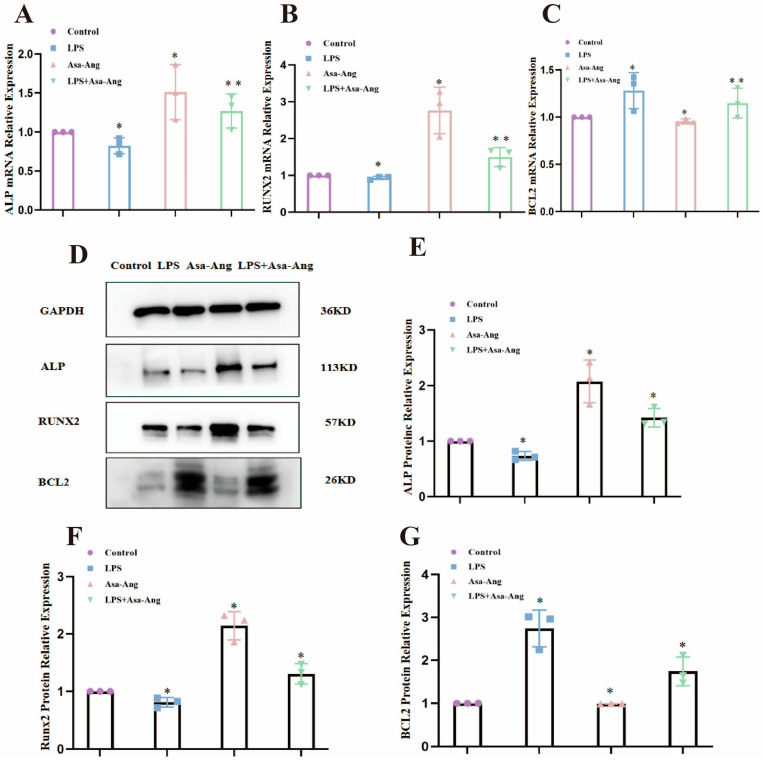
Effect of the Asarum–Angelica drug pair on the expression of ALP, RUNX2, and BCL2 mRNA and protein. (**A**–**C**): Expression of ALP, RUNX2, and BCL2 mRNA by the Asarum–Angelica drug pair. (**D**) Protein bands of Western blot. (**E**–**G**) Expression of ALP, RUNX2, and BCL2 protein by the Asarum–Angelica drug pair (* *p* < 0.05, ** *p* < 0.01).

Real-time qPCR results showed (Figure 6A–C) that the mRNA expression of osteoblast-associated factors ALP, RUNX2, and BCL2 was significantly lower after 24 h of LPS-activated cells compared with the control group (* *p* < 0.05). The Asarum–Angelica drug pair significantly promoted mRNA expression of the above factors (* *p* < 0.05) and promoted mRNA expression of ALP, RUNX2, and BCL2 in the presence of LPS (* *p* < 0.05). The trend in Western blot assay results was consistent with the real-time qPCR results (Figure 6D–G). These results suggest that the Asarum–Angelica drug pair can effectively promote the expression of osteoblast markers ALP, RUNX2, and BCL2 mRNA and protein in an inflammatory environment.

## 3. Discussion

Periodontitis, a localized inflammatory condition, arises from a dysregulated synergy between pathogenic oral microbiota and the host’s immune defenses [6]. This pathology is characterized by the progressive degradation of tooth-supporting structures, including the periodontal ligament and alveolar bone. Clinically, it manifests as gingival inflammation, alveolar bone loss, formation of periodontal pockets, and tooth mobility [26]. The restoration of periodontal bone tissue is vital for successful periodontal intervention. Osteoblast impairment in periodontal tissues contributes to the pathogenesis of bone defects in periodontitis, marked by an increase in osteoclast activity and diminished osteoblast function [27]. Addressing the regeneration of periodontal bone deficits remains a primary objective in dental research. Contemporary strategies often integrate pharmacotherapy with standard periodontal treatments as a conventional modality to combat periodontitis [28]. Evidence suggests that traditional Chinese medicine (TCM) may offer some efficacy in managing periodontitis. The systemic or local adjunctive application of TCM, known for its anti-microbial properties and facilitation of bone remodeling, is garnering increased attention due to its successful application in conditions like osteoporosis and rheumatoid arthritis [29,30].

The treatment of diseases with traditional Chinese medicine has existed for thousands of years. Different to modern medicine, traditional Chinese medicine focuses on adjusting the overall function using traditional Chinese medicine formulas, while modern medicine focuses on local treatment of lesions. Yongqi Zheng, of Yale University, has proposed “We Medicine” as a fusion of Western and Eastern medicinal philosophies. TCM, characterized by its holistic approach, is used for diagnosis, prevention, and treatment, contrasting with Western medicine’s focus on molecular-level targets and single-disease entities [31,32]. Chinese drug pairs are composed of only two types of Chinese medicines, which is the smallest unit and core of the compatibility of traditional Chinese medicine formulas. This is the most common clinical formula form, and the Chinese drug pair has a synergistic effect on efficacy enhancement. Asarum is the dried root and rhizome of Bei Asarum, Seoul Asarum, or Hua Asarum of the Asarum family, first recorded in *Shennong Ben Cao Jing* (Classic of the Divine Husbandman’s Materia Medica). Asarum has the efficacy of dispersing cold, dispelling wind, and relieving pain. It is commonly used clinically to treat wind-cold flu, headache, toothache, nasal congestion and runny nose, and rheumatic paralysis [33]. Angelica is the dried root of the Umbelliferae plant, which is a commonly used bulk medicinal herb in China. It has important effects such as tonifying and activating blood, regulating menstruation, and relieving pain [15]. The Asarum–Angelica drug pair was a common pair of medicines used in the treatment of periodontitis in ancient times. This study employed network pharmacology and combined the “active ingredient target” network to discover that the effective components of the Asarum–Angelica drug pair for the treatment of periodontitis were mainly kaempferol, β-sitosterol, and stigmasterol among 10 active ingredients. Of these, the main active ingredient, kaempferol, can significantly reduce the production of NO induced by *Prevotella intermedia* LPS [34]; reduce alveolar bone resorption, attachment loss, and the production of matrix metalloproteinases MMP-1 and MMP-8 in periodontitis [35]; and promote the proliferation and osteogenic differentiation of periodontal ligament stem cells through the Wnt/β-catenin signaling pathway [36]. β-sitosterol can significantly repress bone marrow-derived macrophage M1 polarization, augment M2 polarization, and attenuate rheumatoid inflammation in mice [37]. β-sitosterol is an anti-inflammatory drug that can participate in regulating leukocyte chemotaxis, increasing vascular permeability, promoting the release of inflammatory mediators from mast cells, and smooth muscle cell contraction, with all these activities contributing to the inhibition of tissue damage caused by inflammation [38]. The anti-inflammatory effects of stigmasterol include producing anti-inflammatory cytokines, reducing the release of inflammatory mediators, and inhibiting the induction of inducible nitric oxide synthase (iNOS) and cyclooxygenase-2 (COX-2) [39]. Stigmasterol exerts anti-diabetic effects by reducing fasting blood glucose, serum insulin levels, and oral glucose tolerance [40].

It was discovered through network graph analysis that targets such as PTGS2, BCL2, AKT1, and CASP3 are the main targets of the Asarum–Angelica drug pair in the treatment of periodontitis. PTGS2 is specifically expressed in periodontitis and plays an important role in inflammation of gingival tissue as well as alveolar bone destruction. PTGS2 can convert arachidonic acid into PGH2, which further synthesizes PGE2 to regulate the inflammatory response of periodontal tissues in periodontitis [41,42]. The apoptosis of inflammatory cells plays an important role in the regression of acute inflammatory responses; BCL2 is an inhibitor of cell apoptosis [43,44,45], and its overexpression during periodontitis exacerbates inflammation. Apart from suppressing cell apoptosis, the BCL2 protein family also plays important roles in cellular energy metabolism, mitochondrial function, autophagy, and cell cycle regulation [46]. In this study, BCL2 was selected as the key target for molecular docking with the main active ingredients of the Asarum–Angelica drug pair. The results showed that the main active ingredients of the Asarum–Angelica drug pair had a strong binding ability with BCL2, suggesting that the drug pair may promote inflammatory cell apoptosis and alleviate periodontitis inflammation by interfering with the formation of BCL2.

The results of the KEGG enrichment analysis showed that the treatment of periodontitis by the Asarum–Angelica drug pair mainly involves the AGE–RAGE signaling pathway, NF-κB signaling pathway, JAK/STAT signaling pathway, small cell lung signaling pathway, and so on. Diabetes, as a risk factor for periodontitis, can exacerbate the occurrence and development of periodontitis [47]. Studies have found that advanced glycosylation end products (AGEs) and receptor of AGEs (RAGE) are expressed in the periodontal tissues of diabetic patients and are highly correlated with oral inflammatory diseases [48]. In gingival fibroblasts, accumulation of AGE leads to up-regulation of MMP-1 expression, which further activates the nuclear factor kappa-B (NF-κB) signaling pathway through the AGE–RAGE signaling pathway, causing an increased expression of interleukin 6 (IL-6) and intercellular cell adhesion molecule-1 (ICAM-1) [49,50]. The NF-κB signaling pathway plays an important role in the development of periodontitis, in which ROS are overproduced, thereby activating the NF-κB signaling pathway, which increases the secretion of pro-inflammatory cytokines and chemokines and regulates the development of inflammation. On the other hand, the NF-κB signaling pathway mediates RANK ligand-induced osteoclastogenesis, which is involved in alveolar bone destruction [50,51]. The JAK/STAT pathway is crucial to many responses, like hematopoiesis, oncogenesis, and immune/inflammation regulation. The JAK3 antagonist CP-690550 showed potential benefits in the treatment of rheumatoid arthritis [52]. It is now clear that the JAK/STAT pathway has a fundamental role in inflammatory diseases onset and progression. This pathway can affect the expression of various genes with pro-inflammatory and anti-inflammatory activity. The enrichment analysis of the KEGG pathway in this study indicated that the combination of Asarum and Angelica can regulate multiple signal pathways involving PTGS2, BCL2, AKT1, and others associated with AGE–RAGE, cancer, JAK/STAT, small cell lung, NF-κB, etc. This suggests that the Asarum–Angelica drug pair may be effective in treating periodontitis in diabetic patients.

ALP is an osteogenic specific factor that regulates bone morphogenesis and is a marker of bone formation [53]. RUNX2 is an early marker of osteoblasts, which reflects the differentiation ability of osteoblasts. In addition, RUNX2 can guide the directed differentiation, transcription, and translation of related osteogenic genes in BMSCs [54]. In this study, LPS was used to act on osteoblastic precursor cells to construct an inflammatory microenvironment, and it was found that the Asarum–Angelica drug pair can effectively reverse the expression of ALP and RUNX2 genes and proteins in the inflammatory microenvironment, indicating that the drug pair has the effect of promoting bone formation in the inflammatory microenvironment. Under the action of LPS, the high expression of BCL2, which can inhibit cell apoptosis, was inhibited. The Asarum–Angelica drug pair can effectively inhibit the expression of BCL2 in the inflammatory microenvironment, thereby promoting inflammatory cell apoptosis. This result is consistent with the molecular docking results in this study, further clarifying the effect of the Asarum–Angelica drug pair on inhibiting the expression of BCL2 during periodontitis and inhibiting inflammatory responses.

A limitation of this study is the reliance on in vitro cellular assays for results validation, with requisite in vivo and clinical trials pending. Therefore, the mechanistic understanding of the Asa–Ang herb pair in periodontitis treatment necessitates further empirical scrutiny.

## 4. Materials and Methods

### 4.1. Screening of Active Components and Target Proteins

The keywords “Asarum” and “Angelica” were submitted to the Traditional Chinese Medicine Systematic Pharmacology Database and Analysis Platform (TCMSP) [55,56], and the unselected active ingredients were added according to the published literature. The active ingredients were screened according to two pharmacokinetic parameters (ADME) of oral bioavailability (OB) ≥ 30% and drug-likeness (DL) ≥ 0.18, and the targets of the active ingredients were predicted using the target prediction model of TCMSP.

### 4.2. Periodontitis-Related Targets Screening

Using the keyword “periodontitis”, the relevant therapeutic targets for the disease in the Genecards database, the DisGeNET database [57,58], the Therapeutic Target Database (TTD), and the Drugbank database were identified [59]. The four database targets were combined and duplicate values were removed, normalized in the Uniport database, and corrected to the gene names of the targets. The Venn diagram package was used to plot Venn diagrams of the obtained active ingredient targets of the Asarum–Angelica drug pair with the potential targets of periodontitis to obtain intersecting sets of common targets (genes), thus obtaining preliminary targets of the Asarum–Angelica drug pair for the treatment of periodontitis.

### 4.3. Network Construction and Correlation Analysis

The software Cytoscape 3.7.1 was used to construct the “active ingredient target” network of Asarum–Angelica. The “node” represents the component or target and the “edge” represents the interaction between the active ingredient and the target; the network characteristics, including degree of connectivity, betweenness, and closeness, were analyzed using the Network Analyzer analysis tool to investigate the relationship between the active ingredient and the target and between the two. In addition, the larger the degrees of freedom and the number of mediators of a node, the more important the node is in the network. The STRING 11.0 database was used for protein interaction analysis of the intersecting targets [60], setting the biological species as “Homo sapiens” and the minimum interaction threshold as “highest confidence > 0.7” to hide the free targets and obtain a protein–protein interaction (PPI) network with high confidence. The “string.tsv” file was downloaded, imported into Cytoscape 3.7.1, topology analysis was performed, Degree was used as node size and color indicator, and the scores for edge thickness and color indicator were combined to draw the PPI network diagram.

### 4.4. GO Enrichment Analysis and KEGG Pathway Analysis

Functional and pathway enrichment analysis was conducted on the intersection of disease and drug targets using the Metascape platform [61]. The biological species was set as “Homo sapiens” and the significance level was set as *p* < 0.01. The main biological processes and metabolic pathways were analyzed and the results were visually presented.

### 4.5. Constituent–Periodontitis Target–Pathway Network Diagram

Cytoscape 3.7.1 was used to construct a network diagram of the Asarum–Angelica pairs–periodontitis target–pathway, analyze the network topology parameters of active ingredients and targets, derive the results, and determine the core targets and the main active ingredients that exert drug effects according to degree, betweenness, and closeness.

### 4.6. Molecular Docking

For the top four targets in the degree-ranked network diagram of the Asarum–Angelica pairs–periodontitis targets obtained from the analysis, AutoDock Vina 1.1.2 software was used to align these targets with the top four key drug active ingredients in the degree-ranked network diagram of the Asarum–Angelica pairs–periodontitis targets. The 3D structure of the protein was obtained with the help of the PDB database and the 3D structure of the drug was obtained using the TCMSP database as molecular docking ligands. The target proteins were first de-watered and de-liganded using PyMOL software; molecular docking studies were then performed using AutoDockTools 1.5.6 software to evaluate the binding activity between the components and the target by the magnitude of the binding energy and were analyzed as graphs using PyMOL [62].

### 4.7. Molecular Dynamics Simulation

To assess the stability of protein–ligand complexes from molecular docking analysis, we used the GPU-accelerated GROMACS 2020 software for MD simulations. A cubic box with 1.0 nm between the solute and the edge of the box was used to build a system for each pair. The system was solvated using the TIP3P water model followed by adding several Na^+^ or Cl^−^ for neutralization. Energy minimization was then performed using the steepest descent algorithm with a maximum of 50,000 steps and <10.0 kJ/mol force parameters. In NVT equilibration, constant temperature (310 K) and volume were established applying a modified Berendsen thermostat algorithm with leap-frog integrator, LINCS holonomic constraints, and Particle Mesh Ewald for long-range electrostatics. Isobaric ensemble was set to maintain a target temperature of 310 K and a target pressure of 1.0 bar in the system. The energy-minimized and -equilibrated systems were subjected to 100 ns atomistic MD simulations.

### 4.8. Preparation Method for the Medicinal Solution of the Asarum–Angelica Herb Pair

To prepare the solution, measure 4 g Asarum and 16 g Angelica. Next, add 10 times the volume of distilled water to the mixture. Allow the mixture to soak for 2 h, followed by bringing it to a boil using high heat. Decoct the solution for two separate intervals of 30 min each. Once the decoction process is complete, allow the solution to cool to room temperature. Finally, precipitate the solution by adding 95% ethanol. The supernatant was obtained through centrifugation, followed by decoction and subsequent concentration to a density of 1 g/mL. The bacteria were then filtered using a microporous membrane, and the resulting solution was stored as a stock solution in sterile bottles. To maintain its integrity, the stock solution was held at a temperature of 4 °C in a sealed container, protected from light.

### 4.9. CCK-8 Assay

Screening of the optimal drug concentration in cells for the action of the Asarum–Angelica drug pair by CCK-8 (Boster, Wuhan, China) was performed. MC3T3-E1 cells were digested and passaged, and then inoculated into 96-well plates at 4 × 10^3^ cells/well, and after the cells were attached to the wall, the original culture medium was discarded. The cells were incubated for 24 h, 48 h, and 72 h with 0, 1 × 10^−1^, 1 × 10^−2^, 1 × 10^−3^, 1 × 10^−4^, 1 × 10^−5^, 1 × 10^−6^, and 1 × 10^−7^ g/mL of the Asarum–Angelica drug pair medicinal solution, then added to CCK-8 solution (Apexbio, Houston, TX, USA) and incubated for 1 h at 37 °C in 5% CO_2_ incubator, and OD values were determined by an enzyme labeler at 450 nm.

### 4.10. Cell Culture

MC3T3-E1 cells (purchased from Wuhan Prosei Life Technology, Wuhan, China) were incubated in α-MEM complete medium containing 10% fetal bovine serum (Gibco, Carlsbad, CA, USA) and 1% penicillin–streptomycin mixture at 37 °C in a 5% CO_2_ incubator; the cells were observed to reach 80–90% density and then passaged for subsequent experiments. Experimental grouping: Control, Asa–Ang, LPS, and LPS + Asa–Ang. MC3T3-E1 osteoblasts were injected in tiny dishes at a density of 2 × 10^6^ cells/well. After 24 h of cell apposition, fresh culture media of 100 ng/mL lipopolysaccharide (LPS) (Sigma-Aldrich, St. Louis, MO, USA) was added to the LPS group, and the new culture medium was replaced in the other groups. After 24 h of incubation, an equal volume of the Asarum–Angelica medicinal solution was added to the Asa–Ang group and the LPS + Asa–Ang group, respectively, and the cells were collected for analysis after further culture for 72 h. The gene expression levels of B-cell lymphoma-2 (BCL2) and osteoblast-associated factors alkaline phosphatase (ALP) and Runt-related Transcription Factor 2 (RUNX2) were detected by RT-qPCR, and the protein expression levels of these factors were analyzed by Western blot.

### 4.11. Real-Time Quantitative PCR

Total RNA from cells was extracted according to the entire RNA extraction kit instructions (GenePharma, Shanghai, China) to remove genomic DNA, with the procedure carried out according to the teachings of the reverse transcription kit and RT-PCR kit (GenePharma, Shanghai, China). The relative change in mRNA was compared using the 2^−ΔΔCt^ method. The primers for *ALP*, *RUNX2*, *BCL2*, and internal reference gene GAPDH were synthesized by GenePharma, and the primer sequences were as follows: (mouse) GAPDH F AAATGGTGAAGGTCGGTGTG; GAPDH R TGAAGGGGTCGTTGATGG; ALP F GGTCTGTGTTCGTAAGGGTGA; ALP R AGGTCAAGAATAAGGTGTAGTC; Runx2 R TTCTACTCGCTGCACTCGG; Runx2 F GGAGACAACATTTATGACGAACGTC; BCL2 R GGAAGGTAGTGTGTGTGG; and BCL2 F ACTCCACTCTCTGGGTTCTTGG.

### 4.12. Western Blot Analysis

Protein lysis solution was made to lyse the cells, total protein was collected, and the protein content was determined by the BCA method. Protein was sampled according to the measured protein concentration, separated by SDS-PAGE electrophoresis, transferred to a PVDF membrane, blocked with 5% skimmed milk powder for 2 h at room temperature, washed with TBST (10 min × 3 times), incubated with primary antibody, and left at 4 °C overnight: ALP (1:1000 dilution; ABclonal, Wuhan, China); RUNX2 (1:500 dilution; HUABIO, Hangzhou, China); BCL2 (1:500 dilution; ABclonal, China); GAPDH (1:5000 dilution; Bioworld, Nanjing, China). The next day, the membrane was washed with TBST (10 min × 3 times), and a secondary antibody (1:1000 dilution; Bioworld, Nanjing, China) was added and incubated for 0.5 h at 37 °C. The membrane was washed with TBST (10 min × 3 times). A chemiluminescent solution (Thermo Fisher Scientific, Waltham, MA, USA) was produced and exposed. The grayscale values of the strips were analyzed using ImageJ software 1.8.0 and the ratio of each target protein to the grayscale value of GAPDH was calculated.

### 4.13. ALP Staining

Following 7 days of induction using the osteogenic induction solution with the addition of the screened optimal concentration of the Asarum–Angelica drug pair, staining was performed according to the BCIP/NBT alkaline phosphatase chromogenic kit procedure and photographed and recorded by microscopic observation.

### 4.14. Alizarin Red Staining

Alizarin red staining was performed after 21 days of induction using an osteogenic induction solution incorporating the screened optimal concentration of the Asarum–Angelica drug pair. MC2T3-E1 cells were fixed in ethanol for 1 h at room temperature, washed twice with PBS, stained with 2 mL of alizarin red solution (pH 4.2) for 15 min, and then rinsed 5 times with PBS. The formation of calcium nodules was observed by microscopy, photographed, and recorded.

### 4.15. Statistical Analysis

All the above data are presented as the mean ± standard deviation (SD). SPSS 17.0 (SPSS Inc., Chicago, IL, USA) software was used to analyze the data. Comparisons between two groups were performed using Tukey’s test (α = 0.05), while multiple groups were evaluated by one-way analysis of variance (ANOVA). The differences were considered statistically significant at *p* < 0.05.

## 5. Conclusions

This study analyzed and validated the main active ingredients, gene targets, and core mechanisms of the action of the Asarum–Angelica drug pair in the treatment of periodontitis through network pharmacology and cell experiments. The results showed that the combination of Asarum and Angelica can act on multiple targets and proteins involved in inflammation, oxidative stress, and apoptosis through a variety of chemical compounds. This reflects the characteristics of traditional Chinese medicine in treating diseases through multiple components, multiple pathways, and multiple targets, offering new directions for the treatment of complex periodontitis of uncertain etiology.

## Figures and Tables

**Table 1:** Main active ingredients of the “Asarum–Angelica” drug pair.

Medicine	Molecular ID	Sign	Main Active Ingredient	OB (%)	DL
Asarum	MOL012140	Asa1	4,9-diamethoxy-1-vinyl-Sb-carboline	65.3	0.19
Asarum	MOL012141	Asa2	Caribine	37.06	0.83
Asarum	MOL001460	Asa3	Cryptopine	78.74	0.72
Asarum	MOL001558	Asa4	Sesamin	56.55	0.83
Asarum	MOL002501	Asa5	[(1S)-3-[(E)-but-2-enyl]-2-methyl-4-oxo-1-cyclopent-2-enyl](1R,3R)-3-[(E)-3-methoxy-2-methyl-3-oxoprop-1-enyl]-2,2-dimethylcyclopropane-1-carboxylate	62.52	0.31
Asarum	MOL002962	Asa6	(3S)-7-hydroxy-3-(2,3,4-trimethoxyphenyl)chroman-4-one	48.23	0.33
Asarum	MOL000422	Asa7	Kaempferol	41.88	0.24
Asarum	MOL003849	Asa8	ZINCO5223929	31.57	0.83
Angelica	MOL000358	Ang1	Beta-sitosterol	36.91	0.75
Angelica	MOL000449	Ang2	Stigmasterol	43.83	0.76

**Table 2:** Results of pathway enrichment of the Asarum–Angelica drug pair on regulating periodontitis targets.

Pathway (Description)	*p* Value	Count	Hits
AGE–RAGE signaling pathway in diabetic complications	−24.2	14	AKT1.BAX.BCL2.CASP3.ICAM1.JUN.NOS3.MAPK8.RELA.SELE.STAT1.TGFB1.TNF.VCAM1
Toxoplasmosis	−23.3	14	AKT1.ALOX5.BCL2.CASP3.CASP8.CASP9.IL10.NOS2.PIK3CG.MAPK8.RELA.STAT1.TGFB1.TNF
Pathways in cancer	−22.5	20	AKT1.AR.BAX.BCL2.CASP3.CASP8.CASP9.GSTM1.HMOX1.IGFIR.JUN.MMP1.NOS2.PPARG.MAPK8.PTGS2.RELA.RXRA.STAT1.TGFB1
Small cell lung cancer	−13.7	9	AKT1.BAX.BCL2.CASP3.CASP9.NOS2.PTGS2.RELA.RXRA
Chemical carcinogenesis	−10.5	7	CHRNA7.CYP1A1.CYP1A2.CYP3A4.GSTM1.PTGS2.UGT1A1
NF-kappa B signaling pathway	−10.1	7	BCL2.ICAM1.PLAU.PTGS2.RELA.TNF.VCAM1
Malaria	−10.1	6	ICAM1.IL10.SELE.TGFB1.TNF.VCAM1
Proteoglycans in cancer	−6.3	6	AKT1.CASP3.IGF1R.PLAU.TGFB1.TNF
Transcriptional misregulation in cancer	−6.3	6	BAX.IGF1R.PLAU.PPARG.RELA.RXRA
Serotonergic synapse	−6.1	5	ALOX5.CASP3.PTGS1.PTGS2.SLC6A4
Cholinergic synapse	−4.6	4	AKT1.BCL2.CHRNA7.PIK3CG
JAK/STAT signaling pathway	−4.1	4	AKT1.BCL2.IL1O.STAT1
Calcium signaling pathway	−3.5	4	ADRB1.CHRNA7.NOS2.NOS3
Dilated cardiomyopathy	−3.5	3	ADRB1.TGFB1.TNF
Proton processing in endoplasmic reticulum	−2.8	3	BAX.BCL2.MAPK8

**Table 3:** Parameters characterizing the nodes of the network of the main active ingredients of the Asarum–Angelica drug pair on the targets of periodontitis.

Target	Degree	Betweenness	Closeness
PTGS2	14	0.16720112	0.5015873
BCL2	10	0.0252735	0.37889688
AKT1	8	0.01028441	0.34422658
CASP3	8	0.01438465	0.37529691
BAX	7	0.01464918	0.37176471
RELA	7	0.00593661	0.3412527
TGFB1	7	0.01337828	0.32985386
TNF	7	0.01206757	0.3412527
RXRA	7	0.01445274	0.34573304
PTGS1	7	0.03150299	0.43051771
SCN5A	7	0.06019114	0.39598997
NOS3	6	0.04788234	0.38820639
NOS2	6	0.01156025	0.35827664

## Data Availability

All data used in this study are included in this article.
